# 6,8-Dichloro-*N*-methyl-3-nitro-4-nitro­methyl-4*H*-chromen-2-amine

**DOI:** 10.1107/S1600536811009366

**Published:** 2011-03-15

**Authors:** J. Muthukumaran, A. Parthiban, M. Kannan, H. Surya Prakash Rao, R. Krishna

**Affiliations:** a Centre for Bioinformatics, Pondicherry University, Puducherry 605 014, India; b Department of Chemistry, Pondicherry University, Puducherry 605 014, India

## Abstract

In the title compound, C_11_H_9_Cl_2_N_3_O_5_, the dihydro­pyran ring adopts a near-half-chair conformation. The benzene ring makes a torsion angle of 5.02 (5)° with the dihydro­pyran ring. Adjacent mol­ecules are inter­linked through inter­molecular C—H⋯O, N—H⋯O and C—Cl⋯π [3.4743 (9) Å] inter­actions. The inter­molecular N—H⋯O hydrogen bond generates an *R*_2_^2^(12) motif, which is observed to contribute to the crystal packing stability. Moreover, the mol­ecular structure displays an *S*(6) motif formed by intra­molecular N—H⋯O hydrogen bonding.

## Related literature

For related structures, see: Gayathri *et al.* (2006 ▶); Bhaskaran *et al.* (2006 ▶). For the biological importance of 4*H-*chromene derivatives, see: Cai (2007 ▶, 2008 ▶); Cai *et al.* (2006 ▶); Gabor (1988 ▶); Brooks (1998 ▶); Valenti *et al.* (1993 ▶); Hyana & Saimoto (1987 ▶); Tang *et al.* (2007 ▶). For ring-puckering analysis, see: Cremer & Pople (1975 ▶).
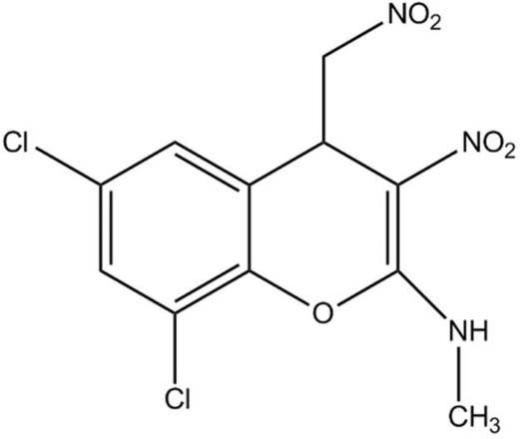


## Experimental

### 

#### Crystal data

C_11_H_9_Cl_2_N_3_O_5_
*M* *_r_* = 334.11
Triclinic, 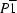

*a* = 8.7426 (7) Å
*b* = 9.2727 (7) Å
*c* = 9.3420 (7) Å
α = 70.017 (7)°
β = 72.609 (7)°
γ = 87.579 (6)°
*V* = 677.68 (9) Å^3^
*Z* = 2
Mo *K*α radiation
μ = 0.50 mm^−1^
*T* = 293 K
0.4 × 0.35 × 0.2 mm

#### Data collection

Oxford Diffraction Xcalibur Eos diffractometer
Absorption correction: multi-scan (*CrysAlis PRO*; Oxford Diffraction, 2009 ▶) *T*_min_ = 0.792, *T*_max_ = 1.000
15150 measured reflections
2385 independent reflections
2072 reflections with *I* > 2σ(*I*)
*R*_int_ = 0.034

#### Refinement

*R*[*F* ^2^ > 2σ(*F* ^2^)] = 0.032
*wR*(*F* ^2^) = 0.110
*S* = 1.01
2385 reflections
191 parameters
H-atom parameters constrained
Δρ_max_ = 0.34 e Å^−3^
Δρ_min_ = −0.30 e Å^−3^

### 

Data collection: *CrysAlis CCD* (Oxford Diffraction, 2009 ▶); cell refinement: *CrysAlis RED* (Oxford Diffraction, 2009 ▶); data reduction: *CrysAlis RED*; program(s) used to solve structure: *SHELXS97* (Sheldrick, 2008 ▶); program(s) used to refine structure: *SHELXL97* (Sheldrick, 2008 ▶); molecular graphics: *ORTEP-3 for Windows* (Farrugia, 1997 ▶) and *PLATON* (Spek, 2009 ▶); software used to prepare material for publication: *PLATON*.

## Supplementary Material

Crystal structure: contains datablocks I, global. DOI: 10.1107/S1600536811009366/zq2089sup1.cif

Structure factors: contains datablocks I. DOI: 10.1107/S1600536811009366/zq2089Isup2.hkl

Additional supplementary materials:  crystallographic information; 3D view; checkCIF report

## Figures and Tables

**Table 1:** Hydrogen-bond geometry (Å, °)

*D*—H⋯*A*	*D*—H	H⋯*A*	*D*⋯*A*	*D*—H⋯*A*
N1—H1⋯O2	0.86	2.00	2.613 (2)	128
N1—H1⋯O2^i^	0.86	2.12	2.881 (2)	147
C7—H7⋯O3^ii^	0.98	2.50	3.1944 (19)	128
C11—H11*B*⋯O3^ii^	0.97	2.54	3.103 (2)	117

Symmetry codes: (i) 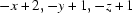; (ii) 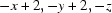.

**Fig. 1.**
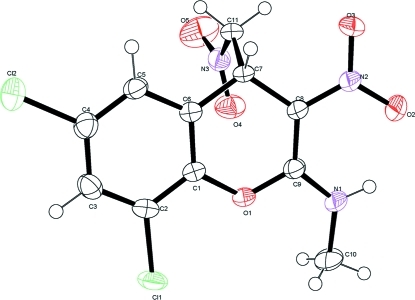
The molecular structure of (I), showing the atom-numbering scheme and displacement ellipsoids drawn at the 50% probability level.

**Fig. 2.**
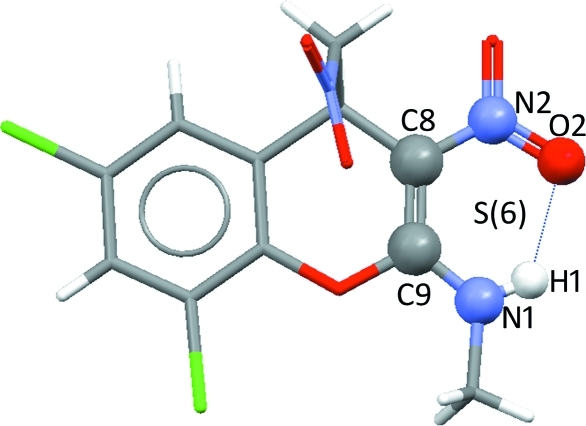
A view of intramolecular motif S (6) formed by N—H···O interaction in (I). The motif forming atoms are shown in ball and stick model and the Hydrogen bond are shown in blue dashed lines.

**Fig. 3.**
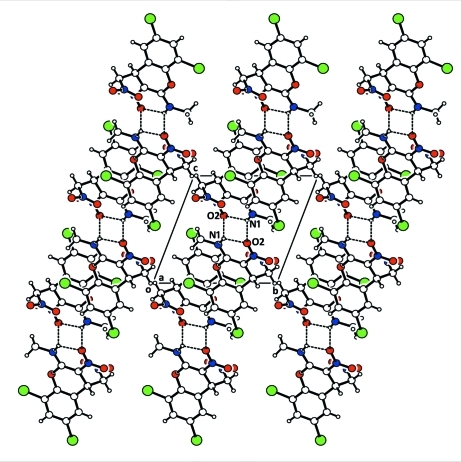
The crystal packing of (I) viewed down the XO-axis, showing intermolecular hydrogen bonding interactions as dashed lines.

**Fig. 4.**
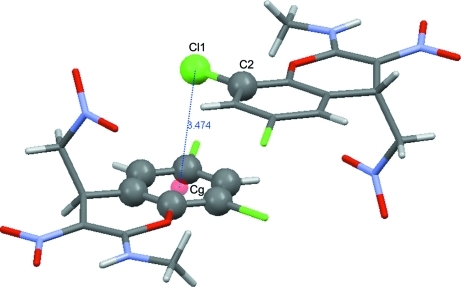
The molecular interaction showing the weak C—Cl···pi interaction in (I). Cg is a centroid of C1—C6 ring in 4H-chromene moiety.

**Fig. 5.**
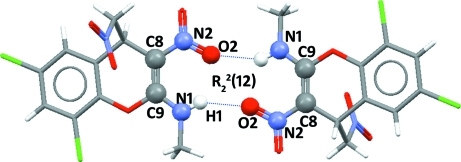
A view of intermolecular ring motif R22 (12) formed by N—H···O interaction in (I). The motif forming atoms are shown in ball and stick model and the hydrogen bond are shown in blue dashed lines.

## supplementary crystallographic information

### Comment

4*H*-Chromenes are biologically important compounds used as synthetic ligands for drug designing and discovery process. They exhibit numerous biological and pharmacological properties such as anti-viral, anti-fungal, anti-inflammatory, anti-diabetic, cardionthonic, anti-anaphylactic and anti-cancer activity (Cai, 2008; Cai, 2007; Cai *et al.*, 2006; Gabor *et al.*, 1988; Brooks, 1998; Valenti *et al.*, 1993; Hyana & Saimoto, 1987; Tang *et al.*, 2007). In view of the growing medicinal importance of 4*H*-chromene derivatives, a single-crystal X-ray diffraction study on the title compound was carried out and analyzed.

The title compound (Fig. 1) contains the 4*H*-chromene moiety with four different substituents [–Cl_2_, –NO_2_, –CH_2_NO_2_ and –NHCH_3_]. The Cl1 group attached to C2 by an (+) anti-periplanar conformation with the torsion angle (Cl1/C2/C3/C4) of 178.76 (14) °, whereas another chlorine attached to C4 with the torsion angle (Cl2/C4/C3/C2) of -176.94 (14) °, which oriented in (-) anti-periplanar conformations. From the puckering analysis (Cremer & Pople, 1975), the fused dihydropyran ring (O1/C1/C6/C7/C8/C9) of 4*H*-chromene is very similar to half chair (H form) conformation with puckering parameters of Q = 0.1772 (17) Å, θ = 104.5 (5) ° and Φ = 11.6 (6) °. The molecular structure is stabilized by intramolecular N—H···O and C—H···O interactions. The intramolecular N1—H1···O2 interaction generates a graph-set motif *S* (6) (Fig. 2) with a *D*···*A* bond distance of 2.613 (2) Å. The crystal packing of the molecule (Fig. 3) is stabilized by intermolecular N1—H1···O2 (symmetry code: -*x* + 2, -*y* + 1, -*z* + 1), C7—H7···O3 (symmetry code: -*x* + 2, -*y* + 2, -*z*), C11—H11B···O3 (symmetry code:-*x* + 2, -*y* + 2, -*z*) and C—Cl··· π (symmetry code: 1 - *x*, 1 - *y*, -*z*) interactions (Fig. 4). The intermolecular N1—H1···O2 interaction generates a ring of graph-set *R^2^_2_* (12) with the bond distance of 2.881 (2) Å (Fig. 5).

### Experimental

*(E)*-2,4-Dichloro-6-(2-nitrovinyl)phenol (100 mg, 0.427 mmol) was taken in a 25 ml round bottom flask in methanol (4 ml). To this solution, 1,8-diazabicyclo[5.4.0] undec-7-ene (DBU) (8 mg, 0.042 mmol) was added and stirred thoroughly for 10 minutes at room temperature. To this stirred solution, NMSM ((*E*) *N*-methyl-1-(methylthio)-2-nitroethenamine) was added and stirred for 10 h for completion (TLC, hexane: EtoAc, 3:2, *R*_f_ of I = 0.3). The reaction mixture was then kept in a refrigerator for 2 h to afford racemic mixture of the product (I), white precipitate, which was filtered. Good crystals were obtained by recrystallization with a solution of dichloromethane: hexane (9:3 *v*/*v*).

### Refinement

All hydrogen atoms were placed in calculated positions, with N—H=0.86 and C—H=0.97 and included in the final cycles of refinement using a riding model with *U*_iso_(H) = 1.2 *U*_eq_(*C*).

### Crystal data

**Table:**

C_11_H_9_Cl_2_N_3_O_5_	*Z* = 2
*M**_r_* = 334.11	*F*(000) = 340
Triclinic, *P*1	*D*_x_ = 1.637 Mg m^−^^3^
Hall symbol: -P 1	Melting point: 485.65 K
*a* = 8.7426 (7) Å	Mo *K*α radiation, λ = 0.71073 Å
*b* = 9.2727 (7) Å	Cell parameters from 8735 reflections
*c* = 9.3420 (7) Å	θ = 2.7–29.2°
α = 70.017 (7)°	µ = 0.50 mm^−^^1^
β = 72.609 (7)°	*T* = 293 K
γ = 87.579 (6)°	Block, colourless
*V* = 677.68 (9) Å^3^	0.4 × 0.35 × 0.2 mm

### Data collection

**Table:**

Oxford Diffraction Xcalibur Eos diffractometer	2385 independent reflections
Radiation source: fine-focus sealed tube	2072 reflections with *I* > 2σ(*I*)
graphite	*R*_int_ = 0.034
Detector resolution: 15.9821 pixels mm^-1^	θ_max_ = 25.0°, θ_min_ = 2.7°
ω scans	*h* = −10→10
Absorption correction: multi-scan (*CrysAlis PRO*; Oxford Diffraction, 2009)	*k* = −11→11
*T*_min_ = 0.792, *T*_max_ = 1.000	*l* = −11→11
15150 measured reflections	

### Refinement

**Table:**

Refinement on *F*^2^	Primary atom site location: structure-invariant direct methods
Least-squares matrix: full	Secondary atom site location: difference Fourier map
*R*[*F*^2^ > 2σ(*F*^2^)] = 0.032	Hydrogen site location: inferred from neighbouring sites
*wR*(*F*^2^) = 0.110	H-atom parameters constrained
*S* = 1.01	*w* = 1/[σ^2^(*F*_o_^2^) + (0.091*P*)^2^] where *P* = (*F*_o_^2^ + 2*F*_c_^2^)/3
2385 reflections	(Δ/σ)_max_ = 0.046
191 parameters	Δρ_max_ = 0.34 e Å^−^^3^
0 restraints	Δρ_min_ = −0.30 e Å^−^^3^

### Special details

**Table:**

Geometry. All e.s.d.'s (except the e.s.d. in the dihedral angle between two l.s. planes) are estimated using the full covariance matrix. The cell e.s.d.'s are taken into account individually in the estimation of e.s.d.'s in distances, angles and torsion angles; correlations between e.s.d.'s in cell parameters are only used when they are defined by crystal symmetry. An approximate (isotropic) treatment of cell e.s.d.'s is used for estimating e.s.d.'s involving l.s. planes.
Refinement. Refinement of *F*^2^ against ALL reflections. The weighted *R*-factor *wR* and goodness of fit *S* are based on *F*^2^, conventional *R*-factors *R* are based on *F*, with *F* set to zero for negative *F*^2^. The threshold expression of *F*^2^ > *σ*(*F*^2^) is used only for calculating *R*-factors(gt) *etc*. and is not relevant to the choice of reflections for refinement. *R*-factors based on *F*^2^ are statistically about twice as large as those based on *F*, and *R*- factors based on ALL data will be even larger.

### Fractional atomic coordinates and isotropic or equivalent isotropic displacement parameters (Å^2^)

**Table:**

	*x*	*y*	*z*	*U*_iso_*/*U*_eq_	
Cl1	0.66030 (6)	0.29149 (5)	−0.00059 (6)	0.04277 (19)	
Cl2	0.68824 (8)	0.83939 (6)	−0.46350 (6)	0.0599 (2)	
O1	0.77375 (15)	0.46548 (12)	0.15081 (14)	0.0344 (3)	
C6	0.79885 (19)	0.71693 (18)	−0.05514 (19)	0.0273 (4)	
O3	1.02216 (15)	0.86894 (13)	0.20553 (15)	0.0389 (3)	
N2	0.98248 (17)	0.72806 (15)	0.25417 (16)	0.0321 (3)	
O2	1.01949 (18)	0.63676 (15)	0.37199 (16)	0.0494 (4)	
O4	0.55005 (17)	0.69199 (16)	0.26437 (17)	0.0502 (4)	
C1	0.7612 (2)	0.55977 (18)	0.00458 (19)	0.0283 (4)	
C5	0.7793 (2)	0.80191 (19)	−0.20214 (19)	0.0312 (4)	
H5	0.8046	0.9075	−0.2450	0.037*	
C7	0.8539 (2)	0.79260 (17)	0.04217 (19)	0.0275 (4)	
H7	0.9526	0.8554	−0.0288	0.033*	
N1	0.8653 (2)	0.40896 (17)	0.35814 (18)	0.0383 (4)	
H1	0.9139	0.4331	0.4152	0.046*	
C3	0.6865 (2)	0.5726 (2)	−0.2272 (2)	0.0365 (4)	
H3	0.6498	0.5253	−0.2850	0.044*	
C8	0.8987 (2)	0.67447 (18)	0.17785 (19)	0.0285 (4)	
C2	0.7067 (2)	0.48781 (19)	−0.0813 (2)	0.0316 (4)	
C9	0.8488 (2)	0.51877 (19)	0.23183 (19)	0.0298 (4)	
N3	0.56967 (19)	0.83127 (18)	0.19612 (18)	0.0373 (4)	
C11	0.7329 (2)	0.90325 (17)	0.0935 (2)	0.0326 (4)	
H11A	0.7759	0.9524	0.1510	0.039*	
H11B	0.7223	0.9831	−0.0017	0.039*	
C4	0.7221 (2)	0.7293 (2)	−0.2847 (2)	0.0355 (4)	
C10	0.8075 (3)	0.2491 (2)	0.4088 (3)	0.0485 (5)	
H10A	0.8584	0.2078	0.3263	0.073*	
H10B	0.6932	0.2433	0.4287	0.073*	
H10C	0.8325	0.1909	0.5049	0.073*	
O5	0.4625 (2)	0.9172 (2)	0.2075 (2)	0.0744 (5)	

### Atomic displacement parameters (Å^2^)

**Table:**

	*U*^11^	*U*^22^	*U*^33^	*U*^12^	*U*^13^	*U*^23^
Cl1	0.0508 (3)	0.0246 (3)	0.0566 (3)	−0.0001 (2)	−0.0175 (2)	−0.0172 (2)
Cl2	0.0942 (5)	0.0534 (4)	0.0365 (3)	0.0011 (3)	−0.0335 (3)	−0.0086 (2)
O1	0.0450 (7)	0.0202 (6)	0.0367 (6)	−0.0035 (5)	−0.0182 (5)	−0.0025 (5)
C6	0.0271 (8)	0.0246 (8)	0.0290 (8)	0.0025 (6)	−0.0077 (6)	−0.0084 (6)
O3	0.0469 (8)	0.0250 (7)	0.0444 (7)	−0.0074 (5)	−0.0186 (6)	−0.0064 (5)
N2	0.0362 (8)	0.0250 (8)	0.0314 (7)	−0.0018 (6)	−0.0139 (6)	−0.0018 (6)
O2	0.0678 (10)	0.0365 (8)	0.0478 (8)	−0.0027 (7)	−0.0388 (7)	−0.0004 (6)
O4	0.0407 (8)	0.0409 (8)	0.0602 (9)	−0.0049 (6)	−0.0082 (7)	−0.0117 (7)
C1	0.0295 (9)	0.0229 (8)	0.0299 (8)	0.0040 (6)	−0.0083 (7)	−0.0068 (6)
C5	0.0348 (9)	0.0245 (9)	0.0293 (8)	0.0015 (7)	−0.0071 (7)	−0.0053 (7)
C7	0.0308 (9)	0.0186 (8)	0.0295 (8)	−0.0006 (6)	−0.0096 (7)	−0.0030 (6)
N1	0.0483 (9)	0.0252 (8)	0.0387 (8)	−0.0021 (6)	−0.0221 (7)	0.0006 (6)
C3	0.0392 (10)	0.0387 (11)	0.0383 (10)	0.0041 (8)	−0.0126 (8)	−0.0208 (8)
C8	0.0309 (9)	0.0229 (8)	0.0307 (8)	0.0002 (7)	−0.0130 (7)	−0.0044 (6)
C2	0.0309 (9)	0.0238 (9)	0.0401 (9)	0.0028 (7)	−0.0077 (7)	−0.0136 (7)
C9	0.0296 (9)	0.0248 (9)	0.0322 (9)	0.0021 (7)	−0.0109 (7)	−0.0050 (7)
N3	0.0405 (9)	0.0388 (9)	0.0378 (8)	0.0085 (7)	−0.0150 (7)	−0.0177 (7)
C11	0.0397 (10)	0.0210 (8)	0.0383 (9)	0.0022 (7)	−0.0163 (7)	−0.0078 (7)
C4	0.0409 (10)	0.0369 (10)	0.0277 (8)	0.0045 (8)	−0.0101 (7)	−0.0106 (7)
C10	0.0579 (13)	0.0245 (10)	0.0514 (11)	−0.0055 (9)	−0.0192 (10)	0.0047 (8)
O5	0.0539 (10)	0.0656 (11)	0.0901 (13)	0.0263 (9)	−0.0061 (9)	−0.0262 (9)

### Geometric parameters (Å, °)

**Table:**

Cl1—C2	1.7287 (16)	C7—H7	0.9800
Cl2—C4	1.7391 (17)	N1—C9	1.311 (2)
O1—C9	1.352 (2)	N1—C10	1.455 (2)
O1—C1	1.3812 (19)	N1—H1	0.8600
C6—C1	1.385 (2)	C3—C2	1.381 (2)
C6—C5	1.388 (2)	C3—C4	1.379 (2)
C6—C7	1.509 (2)	C3—H3	0.9300
O3—N2	1.2537 (17)	C8—C9	1.398 (2)
N2—O2	1.2593 (18)	N3—O5	1.208 (2)
N2—C8	1.370 (2)	N3—C11	1.492 (2)
O4—N3	1.222 (2)	C11—H11A	0.9700
C1—C2	1.392 (2)	C11—H11B	0.9700
C5—C4	1.383 (2)	C10—H10A	0.9600
C5—H5	0.9300	C10—H10B	0.9600
C7—C8	1.507 (2)	C10—H10C	0.9600
C7—C11	1.531 (2)		
			
C9—O1—C1	120.56 (13)	N2—C8—C7	116.78 (13)
C1—C6—C5	118.53 (15)	C9—C8—C7	122.28 (14)
C1—C6—C7	119.89 (14)	C3—C2—C1	120.45 (15)
C5—C6—C7	121.54 (14)	C3—C2—Cl1	120.48 (13)
O3—N2—O2	120.24 (13)	C1—C2—Cl1	119.04 (13)
O3—N2—C8	119.38 (12)	N1—C9—O1	111.86 (15)
O2—N2—C8	120.38 (13)	N1—C9—C8	127.76 (16)
O1—C1—C6	123.02 (14)	O1—C9—C8	120.38 (14)
O1—C1—C2	116.01 (14)	O5—N3—O4	123.38 (17)
C6—C1—C2	120.97 (15)	O5—N3—C11	116.64 (16)
C4—C5—C6	119.86 (15)	O4—N3—C11	119.98 (14)
C4—C5—H5	120.1	N3—C11—C7	115.17 (13)
C6—C5—H5	120.1	N3—C11—H11A	108.5
C8—C7—C6	110.98 (13)	C7—C11—H11A	108.5
C8—C7—C11	114.16 (13)	N3—C11—H11B	108.5
C6—C7—C11	111.64 (13)	C7—C11—H11B	108.5
C8—C7—H7	106.5	H11A—C11—H11B	107.5
C6—C7—H7	106.5	C3—C4—C5	121.98 (15)
C11—C7—H7	106.5	C3—C4—Cl2	118.91 (13)
C9—N1—C10	124.73 (17)	C5—C4—Cl2	119.08 (13)
C9—N1—H1	117.6	N1—C10—H10A	109.5
C10—N1—H1	117.6	N1—C10—H10B	109.5
C2—C3—C4	118.19 (15)	H10A—C10—H10B	109.5
C2—C3—H3	120.9	N1—C10—H10C	109.5
C4—C3—H3	120.9	H10A—C10—H10C	109.5
N2—C8—C9	120.80 (14)	H10B—C10—H10C	109.5
			
C9—O1—C1—C6	−10.9 (2)	C4—C3—C2—Cl1	178.69 (13)
C9—O1—C1—C2	169.50 (14)	O1—C1—C2—C3	178.49 (15)
C5—C6—C1—O1	−178.84 (15)	C6—C1—C2—C3	−1.1 (2)
C7—C6—C1—O1	−1.1 (2)	O1—C1—C2—Cl1	0.0 (2)
C5—C6—C1—C2	0.8 (2)	C6—C1—C2—Cl1	−179.62 (13)
C7—C6—C1—C2	178.55 (15)	C10—N1—C9—O1	−1.3 (3)
C1—C6—C5—C4	0.5 (2)	C10—N1—C9—C8	178.82 (18)
C7—C6—C5—C4	−177.25 (15)	C1—O1—C9—N1	−172.63 (14)
C1—C6—C7—C8	14.2 (2)	C1—O1—C9—C8	7.2 (2)
C5—C6—C7—C8	−168.08 (15)	N2—C8—C9—N1	3.4 (3)
C1—C6—C7—C11	−114.39 (16)	C7—C8—C9—N1	−172.13 (17)
C5—C6—C7—C11	63.3 (2)	N2—C8—C9—O1	−176.45 (15)
O3—N2—C8—C9	−178.20 (15)	C7—C8—C9—O1	8.0 (2)
O2—N2—C8—C9	1.9 (2)	O5—N3—C11—C7	−163.02 (15)
O3—N2—C8—C7	−2.4 (2)	O4—N3—C11—C7	17.5 (2)
O2—N2—C8—C7	177.65 (15)	C8—C7—C11—N3	−65.94 (19)
C6—C7—C8—N2	166.39 (14)	C6—C7—C11—N3	60.95 (17)
C11—C7—C8—N2	−66.4 (2)	C2—C3—C4—C5	1.1 (3)
C6—C7—C8—C9	−17.9 (2)	C2—C3—C4—Cl2	−176.92 (14)
C11—C7—C8—C9	109.33 (17)	C6—C5—C4—C3	−1.4 (3)
C4—C3—C2—C1	0.2 (3)	C6—C5—C4—Cl2	176.55 (13)

### Hydrogen-bond geometry (Å, °)

**Table:**

*D*—H···*A*	*D*—H	H···*A*	*D*···*A*	*D*—H···*A*
N1—H1···O2	0.86	2.00	2.613 (2)	128
N1—H1···O2^i^	0.86	2.12	2.881 (2)	147
C7—H7···O3^ii^	0.98	2.50	3.1944 (19)	128
C11—H11B···O3^ii^	0.97	2.54	3.103 (2)	117

Symmetry codes: (i) −*x*+2, −*y*+1, −*z*+1; (ii) −*x*+2, −*y*+2, −*z*.
